# The Effect of Sample Bias and Experimental Artefacts on the Statistical Phylogenetic Analysis of Picornaviruses

**DOI:** 10.3390/v11111032

**Published:** 2019-11-06

**Authors:** Yulia Vakulenko, Andrei Deviatkin, Alexander Lukashev

**Affiliations:** 1 Martsinovsky Institute of Medical Parasitology, Tropical and Vector Borne Diseases, Sechenov First Moscow State Medical University, 119435 Moscow, Russia; vjulia94@gmail.com; 2 Faculty of Biology, Lomonosov Moscow State University, 119234 Moscow, Russia; 3 Institute of Molecular Medicine, Sechenov First Moscow State Medical University, 119048 Moscow, Russia; andreideviatkin@gmail.com

**Keywords:** Bayesian phylogenetics, sample bias, picornaviruses, enterovirus A71

## Abstract

Statistical phylogenetic methods are a powerful tool for inferring the evolutionary history of viruses through time and space. The selection of mathematical models and analysis parameters has a major impact on the outcome, and has been relatively well-described in the literature. The preparation of a sequence dataset is less formalized, but its impact can be even more profound. This article used simulated datasets of enterovirus sequences to evaluate the effect of sample bias on picornavirus phylogenetic studies. Possible approaches to the reduction of large datasets and their potential for introducing additional artefacts were demonstrated. The most consistent results were obtained using “smart sampling”, which reduced sequence subsets from large studies more than those from smaller ones in order to preserve the rare sequences in a dataset. The effect of sequences with technical or annotation errors in the Bayesian framework was also analyzed. Sequences with about 0.5% sequencing errors or incorrect isolation dates altered by just 5 years could be detected by various approaches, but the efficiency of identification depended upon sequence position in a phylogenetic tree. Even a single erroneous sequence could profoundly destabilize the whole analysis by increasing the variance of the inferred evolutionary parameters.

## 1. Introduction

The introduction of statistical phylogenetic methods (also termed Bayesian phylogenetics) over a decade ago allowed the timing of evolutionary events that occurred in the past to be elucidated by applying complex evolutionary and epidemiological models to contemporary sequences [1]. This novel algorithm was especially well-suited for RNA viruses which acquire nucleotide substitutions at high rates, usually in the order of 10^−2^ to 10^−5^ substitutions/site/year (s/s/y) [2]. Picornaviruses have an error-prone replication machinery, and most of them also feature short infection cycles and rarely persist in their hosts, which results in very high substitution rates, even compared to other RNA viruses, usually between 10^−2^ to 10^−3^ (Table 1). As a result, even relatively short sequence fragments produced by Sanger sequencing, often as a part of a surveillance routine, are suitable for statistical phylogenetic analysis.

**Figure 1:**
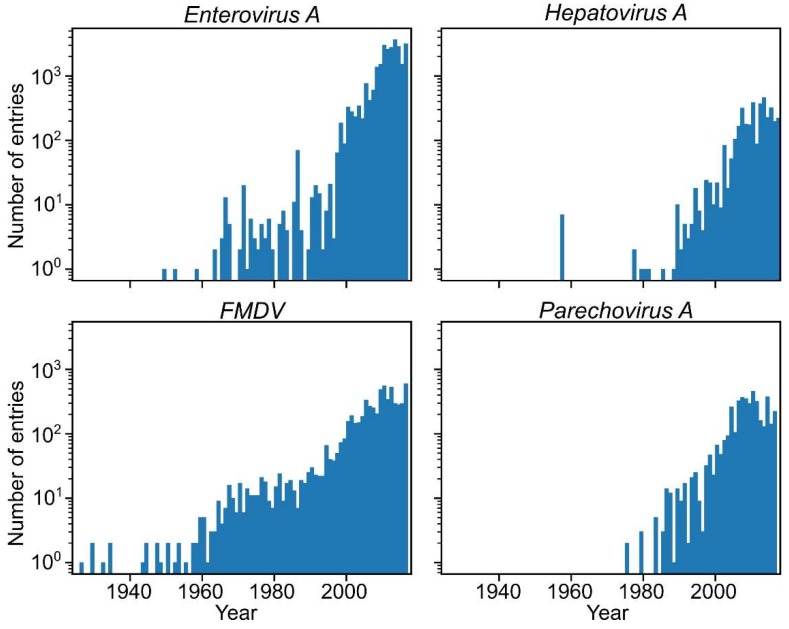
Distribution of collection dates in GenBank entries of the most common picornavirus species—*Enterovirus A*, *Foot-and-mouth disease virus* (*FMDV*), *Hepatovirus A*, and *Parechovirus A*. Numbers of entries are shown on a logarithmic scale.

The value of phylogenetic reconstructions relies upon the selected substitution and population models, as well as the quality and quantity of the sequence data. Selection of calculation parameters appropriate to the data requires careful preliminary analysis and has been well covered in recent publications [17,18,19,20,21]. The preparation of a sequence dataset has received less attention, but its impact is rapidly increasing. The number of available picornavirus sequences has grown exponentially in recent decades (Figure 1). This is both a blessing and a challenge for Bayesian phylogenetic analysis, since the large number of parameters and the model complexity, together with the rising amount of sequence data, have considerably elevated the computational demands. Moreover, the analysis of thousands of virus sequences may be both too long and uninformative because of a lack of phylogenetic resolution (reliably supported groups with high posterior probability values) due to the presence of many identical or indistinguishable sequences [22]. Therefore, the preparation of a dataset for analysis is becoming at least as important as the selection of analysis parameters. Below, we have summarized how the sequence dataset can affect the statistical phylogenetic analysis of picornaviruses.

## 2. Materials and Methods

### 2.1. Distribution of Picornavirus Collection Dates

All sequences above 100 nt available for species *Enterovirus A*, *Parechovirus A*, *Hepatovirus A*, and *Foot-and-mouth disease virus (FMDV)* as of July 2019 were downloaded from the GenBank nucleotide sequence database. The collection years were extracted from the “collection_date” field, and the frequency distribution histogram of collection years was plotted using a Python script.

### 2.2. Distribution of Genome Fragments Deposited in GenBank along the Genome

All available sequences of *Enterovirus A*, *FMDV*, *Hepatovirus A*, and *Parechovirus A* above 100 nt were extracted from GenBank. Several complete genome sequences within each species were then defined as reference sequences. The local BLAST [23] database was created using downloaded sequences, and a blastn search of reference complete genomes was performed against a local database. The coverage of reference sequences by blast hits was summarized for each species. Python scripts used to generate the figure are available at https://github.com/v-julia/Coverage.

### 2.3. Preparation of the EV-A71 Reference Alignment

All available sequences of enterovirus A71 (EV-A71) were obtained from GenBank database in GenBank format. This file was converted into fasta format, sequences below 800 nt and over 8000 nt were omitted. Accession numbers and collection dates were automatically retrieved from the GenBank entries and added to the sequence descriptors. A local blast database was created from downloaded sequences, and a blast search of VP1 encoding gene sequence of the prototype strain BrCr (GenBank number JN874547) was performed against this database. Blast hits were aligned using MAFFT v.7.304 [24]. The full VP1 sequence was excised from the alignment according to the reference VP1 sequence of the prototype strain BrCr. This resulted in an alignment with 7026 sequences. Sequences that lacked information about collection dates in the GenBank annotation or in the corresponding publications were omitted, yielding a dataset of 6902 full VP1 sequences.

### 2.4. Generating Random Sequence Sets from the Reference Alignment

The reference set of EV-A71 full VP1 sequences was used to generate reduced alignments. The following algorithms were applied using a Python script:

- Random sampling of data subsets corresponding to distinct studies (“random groups”). All sequences in the reference alignment were partitioned into groups based on the first five characters of the accession number. A random group was then chosen, and all sequences from this group were added to the alignment. This step was repeated until the number of sequences reached the defined value. This algorithm reproduced the situation with enterovirus sampling 15 years ago, when only a few studies were done, or the currently available sample for less common types and species.
- Random sampling of sequences (“random single”). The selected number of sequences was randomly picked from the initial data set.
- Identity filtration of sequences. Sequences that differed from any other entry in the dataset by less than the selected percentage of the nucleotide sequence were omitted. The comparison of sequences by the script started from the first sequence in the dataset; therefore, the initial alignment was shuffled prior to each repetition of sampling.
- “Smart picking”. All sequences in the initial alignment were partitioned into groups based on the first five characters of the accession number. Sequences from the subsets with a size that did not exceed the user-defined threshold were all included in the final dataset; for bigger subsets, one sequence or a defined fraction of randomly chosen sequences was added to the reduced dataset. This sampling algorithm allowed the inclusion of unique sequences from small studies and reduced the number of sequences from massive epidemiological investigations. For each Genbank number range, at least two sequences were included, or 1% from the larger studies. These conditions were necessary to sufficiently reduce the large EV-A71 dataset; less stringent parameters may be recommended for taxa with fewer studies presented in GenBank.

Ten alignments were generated using the “random groups” algorithm, in order to assess the effect of the number of sequences in alignment on the tree height and substitution rate inferred using Bayesian phylogenetic methods. Ten datasets were generated by each of the other three methods to analyze the effect of sample preparation algorithms on the Bayesian Skygrid reconstruction. 

### 2.5. Bayesian Phylogenetic Analysis

Phylogenetic analysis of the alignments generated was done in a Bayesian statistical framework using a Markov chain Monte Carlo (MCMC) approach implemented in Beast v.1.10 [25]. The HKY model with gamma-heterogeneity among sites and partitioning of sequence data into (first + second) and third codon positions (SRD06 model [26]) was used with the log-normal molecular clock assumption and Bayesian Skygrid model [27], which allows population dynamics to be inferred. Each analysis was performed until the effective sample sizes (ESS) of all parameters reached 200. The number of generations varied between 50 and 150 million generations. MCMC convergence was inspected using Tracer 1.6 [28]. Maximum clade credibility (MCC) trees were annotated with TreeAnnotator v.1.8.2 using a 20% burn-in. Trees were visualized with FigTree v.1.4.2 [29]. Substitution rates, tree heights, and Bayesian Skygrids were visualized using R scripts.

### 2.6. Effect of Sequencing Errors and Errors in Annotation on Evolutionary Estimates

Full VP1 sequences of EV-A71 genotype B1 (*n* = 56) were extracted from the reference alignment. A Python script was used to add 5, 10, or 20 mutations to sequences AB524092 and AF135866. To test the effect of isolation date errors, the dates of AB524092 and AF135866 were decreased or increased by 5, 10, 15, or 20 years. Maximum likelihood (ML) phylogenetic trees were built for the original alignment of genotype B1 and the simulated alignments using IQ-TREE [30], and the temporal structure in each tree was analyzed using TempEst software [31]. The Bayesian phylogenetic analysis of the datasets produced with simulated erroneous sequences was performed using Beast v.1.10 [25], with the same model parameters as described above, except the constant population size, because population dynamics were not relevant for this test. Branch substitution rates were obtained from the annotated MCC tree file and their frequency distribution was drawn using a Python script.

All Python and R scripts used for data preparation and analysis are available at https://github.com/v-julia/sample_bias.

## 3. Results and Discussion

### 3.1. Selection of a Genome Region and Recombination Analysis

**Figure 2:**
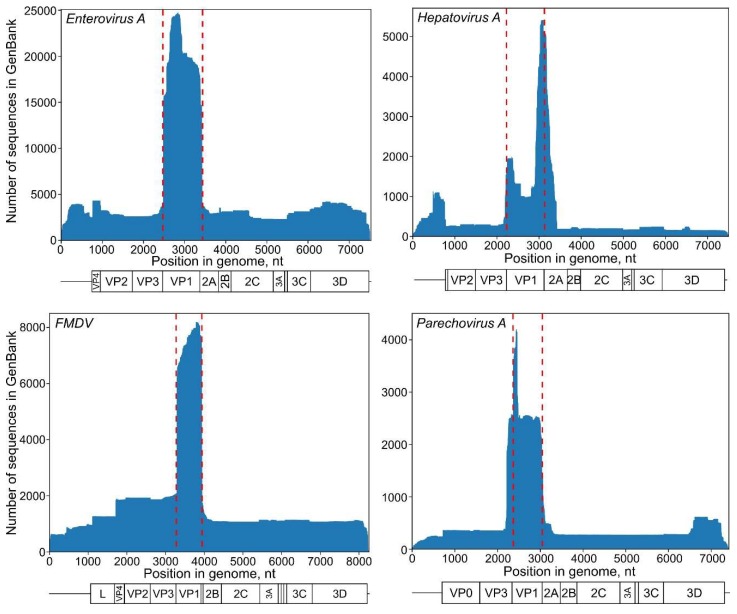
The distribution of genome fragments deposited in GenBank as of July 2019 along the genome in *Enterovirus A*, *Foot-and-mouth disease virus (FMDV)*, *Hepatovirus A*, and *Parechovirus A*. The y-axis indicates the number of sequences for each genome position shown on the *x*-axis. Dashed red lines indicate the position of the VP1 protein-encoding sequence.

Each virus species has regions that are traditionally used for phylogenetic analysis; among picornaviruses, this is usually the VP1 genome region, which encodes for the major capsid protein. VP1 sequences are the most abundant in GenBank for most common picornaviruses (Figure 2). In most cases, VP1 is well-suited to phylogenetic analysis. Other genome regions should be used with care, because recombination, which can compromise phylogenetic analysis, is rare in VP2–VP3–VP1 genome regions, but very frequent elsewhere, including VP4, 2A, 2C, and 3CD in the most common picornavirus genera, such as *Enterovirus*, *Aphthovirus*, *Parechovirus*, and *Cardiovirus* [32,33]. In general, other genome regions should not be used for statistical phylogenetics, unless the absence of recombination has been proven by analysis with a set of algorithms (for example, the RDP4 software package [34]) and by demonstrating congruence between phylogenies for the VP1 gene and a selected genome region. Recombination within the VP2–VP3–VP1 region is very rare, but not impossible [35]. Moreover, there may be GenBank entries with sequencing artefacts that mimic recombination. In the case of statistical phylogenetics, even much less severe sequencing errors can have a profound effect on the whole analysis (see below); therefore, a quick recombination screening would improve the reliability of any study.

In *hepatitis A virus* (*HAV*) and *Aichi virus* (*AiV*), recombination may occur in any genome region [36,37]. In *HAV*, at least, this corresponds to the absence of distinct serotypes [5]. Using the historically abundant VP1-2A junction sequences (Figure 2) is preferable for statistical phylogenetic studies. A recombination analysis is recommended prior to any analysis in these viruses and, since it may be hard to detect ancient or short-length recombination events, this poses an additional challenge for *HAV* and *AiV* studies. 

### 3.2. Selection of a Taxon

A major advantage of statistical phylogenetics is the integrated molecular clock analysis, which also introduces additional requirements to the dataset. Bayesian phylogenetic methods assume the presence of a temporal signal in the sequence dataset, which should be confirmed prior to a study using regression analysis of root-to-tip distances (distances between the tree root and a sequence) and tip dates. This approach was implemented in TempEst software [31]. Temporal signal is usually disrupted by mutation saturation, and overall positive correlation between root-to-tip distances and isolation dates does not assure the absence of mutation saturation at all tree levels, as a strong correlation among the recent isolates may mask incongruencies at the basal tree branches. Mutation saturation can be further assessed using DAMBE software by plotting the number of transitions and transversions against genetic distance, calculation of entropy-based index of substitution saturation, and other methods [38]. Estimates of transition/transversion ratio (ti/tv) have been shown to be a powerful means to investigate whether the chosen substitution model correctly accounts for site saturation, as ti/tv ratio should be constant in time for a virus group [39].

The date randomization test is another way to determine whether the estimates of rates and timescales are reliable. In this method, the tip dates in a dataset are randomized, and Bayesian phylogenetic analysis is performed several times. The rate estimated using the original dataset is considered reliable if it falls outside the 95% confidence interval of the rates of datasets with randomized sampling times [40,41,42].

There are no fossil data available for picornaviruses, as the oldest *FMDV* isolate dates back to 1926, and other genera are even “younger” (Figure 1). Fossil data, such the genomes of phleboviruses integrated into insect genomes, may shift the common ancestor dating by several orders of magnitude [43,44]; therefore, picornavirus root dates should be treated with caution. Attempts to better address mutation saturation in Bayesian phylogenetics are currently underway [45], but have not yet become the mainstream methodology. Thus, a practical recommendation may be to limit the dataset size and diversity according to the study goals and avoid overly broad analysis and interpretation.

Another important consideration for taxon selection is that common evolutionary models in statistical phylogenetics are tailored for population analysis. This limitation is often ignored in real life; moreover, it may be difficult to define a “population” in virology. Due to recombination, parts of the non-segmented picornavirus genome evolve independently [33] and may have distinct population boundaries. In the case of capsid genes, types (a sub-species taxonomic level in most picornaviruses) have properties of global populations. Analysis of several types in a single dataset should be interpreted with great care because the mechanisms responsible for the emergence of types are poorly understood, and may include “quantum evolution” (extremely rapid profound changes) [46], which are poorly compatible with most evolutionary models. Thus, in the structural genes (including VP1), it is again not recommended to analyze several types at once. In the non-structural protein genes, a population corresponds to the species level [33], but, as discussed in Section 3.1, such an analysis would most likely be invalid due to frequent recombination.

### 3.3. Effect of Dataset Size and Sample Bias on Evolutionary Estimates

The number of available sequences exceeds 10,000 for the most relevant picornaviruses but is much smaller for others. Larger datasets allow performing simulations to illustrate the relevance of smaller datasets available for less common species and types. To this end, we conducted a phylogenetic analysis of artificially reduced simulated alignments of the full VP1 genome region (891 nt). It is one of over a hundred of enterovirus types and the most neurovirulent non-polio enterovirus, which caused massive outbreaks of hand-foot-and-mouth disease in Asia [47]. The virus is represented by seven subtypes termed A, B0–B5, C1–C5, D, E, F, and G [13,48], of which subtypes B and C are the most prevalent [49,50]. Due to its medical significance and ubiquitous presence, EV-A71 is the most extensively sequenced enterovirus type and a convenient model for simulation studies.

The representation of isolation locations among sequences of individual taxa available in GenBank is highly heterogeneous. In many cases, the data come from one or a few large studies, some done in a single region. In order to simulate this bias using the EV-A71 dataset, the sequences were added to the simulated datasets by blocks with the identical first five characters of the GenBank accession number, which usually corresponded to a single study, until the dataset had the defined number of sequences. This sampling mode simulated the situation in which only a few studies have been done for a taxon.

A total of 6902 EV-A71 VP1 sequences with known collection dates were available as of July 2019. Sets of 10 alignments containing 50, 100, 200, or 400 sequences were created. This range of sequence numbers corresponds to the datasets available in GenBank for most EV types or picornavirus species.

**Figure 3:**
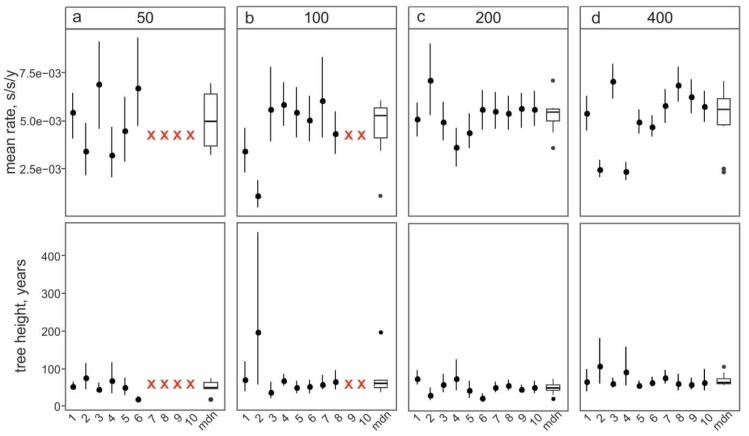
The effect of the number of sequences in the alignment on the substitution rate and root height inferred using Bayesian phylogenetic analysis. The median values and 95% high probability density (HPD) intervals of the mean substitution rate and tree heights are shown as dots and ticks for each of ten sampling replications with the specified number of sequences, respectively. Red crosses indicate runs that failed to converge after 200 million generations. Boxplots show the median values (mdn) and outliers for each panel (**a**–**d**).

Bayesian framework parameters did not converge in four out of ten 50 sequence datasets and two out of ten 100 sequence datasets (Figure 3, red crosses). This apparently happened due to the high prevalence of similar sequences from a single study in the dataset and exemplifies that even a significant number of sequences obtained in just one or few studies (often the case for newly discovered picornaviruses) may be insufficient for Bayesian inference. The variances of substitution rates and tree root ages were huge, even in datasets containing 400 sequences (Figure 3). Substitution rates varied among datasets selected from the same initial pool of sequences between 2.4 × 10^−3^ and 7.0 × 10^−3^ s/s/y even in large datasets of 400 sequences, and tree heights (ages of the most recent common ancestor of EV-A71) could vary between 60 and 105 years, with non-overlapping 95% high probability density (HPD) intervals.

This simulation shows the effect of sample bias, which is inherent to most phylogenetic studies, on the key evolutionary estimates in picornaviruses. As most studies use all available sequence data for a taxon, it is not possible to assess the bias of an available dataset relative to the general population. It would be prudent to assume that the key evolutionary estimates for smaller picornavirus sequence datasets might be significantly affected by sampling bias, in addition to any of the uncertainty which is inherent to statistical phylogenetics itself.

### 3.4. Approaches to Reducing the Dataset

**Figure 4:**
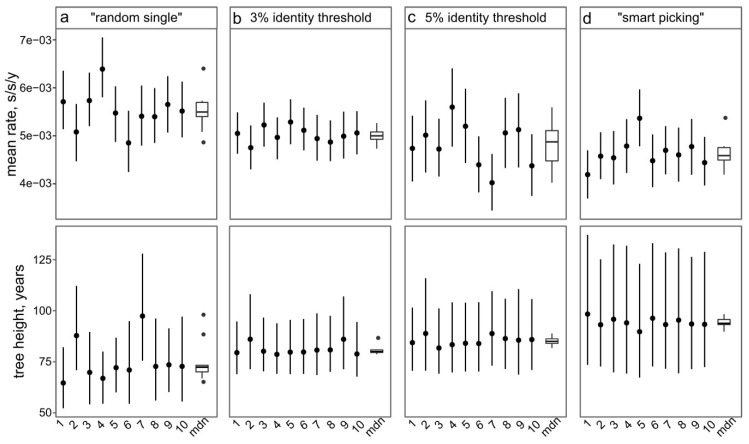
The effect of dataset preparation algorithm on the Bayesian inference of mean substitution rate and tree height. Ten alignments were created from a set of 6902 EV-A71 VP1 sequences using the following algorithms: picking 400 random sequences (**a**); identity filtration with 3% cut-off, resulting in 380 sequences (**b**); and 5% cut-off, resulting in 95 sequences (**c**); and a “smart picking” algorithm, resulting in 447 sequences (**d**). The median values and 95% HPD intervals of the mean substitution rate and tree heights for each of ten sampling replications are shown as dots and ticks, respectively. Boxplots show the median values (mdn) and outliers for each panel.

**Figure 5:**
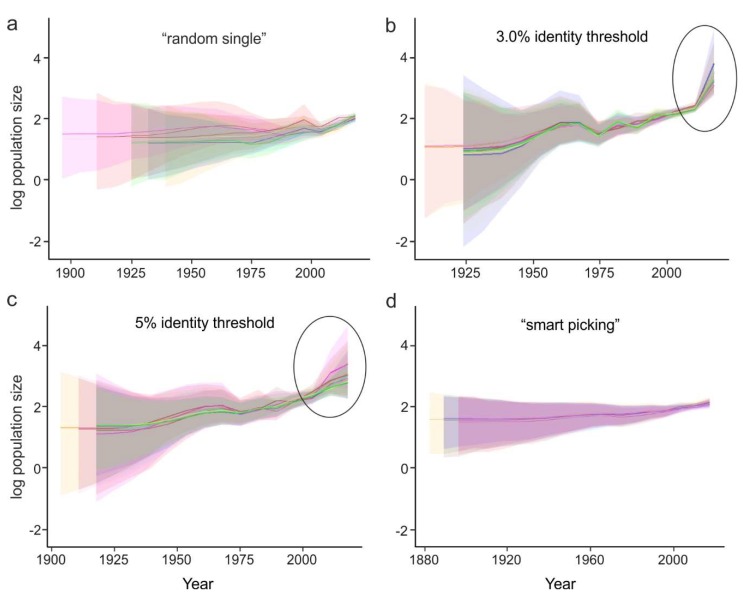
The effect of the dataset preparation algorithm on the results of population dynamics reconstruction using the Bayesian Skygrid model. Random picking of 400 random sequences (**a**), identity filtration with 3% (**b**) and 5% (**c**) identity threshold; “smart picking” algorithm (**d**). Ten repetitions of each sampling algorithm are shown in different colors. Shaded areas indicate 95% high probability density intervals. Similar sequence removal caused a false explosive population growth at a time corresponding to the cut-off threshold (circled).

Analyzing thousands of sequences is both computationally difficult and uninformative. In many cases, it is necessary to reduce the dataset while keeping it as representative as possible. The possible approaches are:

1. Random sampling of sequences. In this case, the sequences from large studies (sometimes originating from narrow samples, such as outbreaks) would be over-represented, while rare (but very informative) sequences may be lost. This results in a significant variation of the key evolutionary estimates (Figure 4a) and the Bayesian estimation of past population dynamics using Bayesian Skygrid analysis (Figure 5a).
2. Identity filtration—discarding sequences that are almost identical to each other. This can be done using Jalview [51], CD-HIT [52], UCLUST [53], the skipredundant tool in EMBOSS software [54], or in-house scripts. This approach ensures that the reduced dataset is as informative in terms of genetic diversity as the original one because all rare sequences are preserved. Identity filtration can be considered in most phylogenetic studies because it is simple and results in a much more limited variation of evolutionary estimates than random picking (Figure 4b). However, an overly stringent reduction can lead to increased deviations of evolutionary estimates (Figure 4c). In the case of statistical phylogenetic studies, identity filtration can introduce bias by itself. Most prominently, it can result in artefacts in the Bayesian Skygrid analysis, because artificial removal of similar sequences universally discards the most recent tree nodes and thus simulates explosive population growth at a time that corresponds to the cut-off threshold (Figure 5b,c, circled).
3. “Smart picking” first identifies the sequences that most likely belong to distinct studies based on the first five characters (two letters and three digits) of the GenBank accession number. All small studies are then included, while datasets from larger studies are reduced by random sampling proportionally to their size. In this way, rare sequences are less likely to be lost, and no bias should be introduced. Indeed, the variation of evolutionary estimates in this case was low (Figure 4d), and no artefacts were apparent in the Skygrid analysis (Figure 5d). This algorithm was implemented using in-house scripts (available at https://github.com/v-julia/sample_bias). This method requires more manual tuning than the identity filtration to obtain the desired dataset size, and overly complicated datasets, such as EV-A71, may be difficult to reduce. However, smart sampling is also less prone to introducing additional bias in population dynamics analysis (Figure 5d in comparison to Figure 5b,c, circled), and produces reproducible population size estimates (Figure 5d).

The choice between the latter two approaches should be made individually based on the study parameters. In any case, the sequence alignment should additionally be manually checked for potential sequencing errors, such as frameshifts in a coding sequence, regions of unnatural heterogeneity in particular sequences, etc., which occur in GenBank and may have a profound effect on the analysis.

### 3.5. Managing Ambiguous Sequence Characters

Picornaviruses exist as quasispecies, and each virus sample contains a cloud of almost identical genomes, which differ by a few substitutions and together make up the master sequence. Sometimes, the relative number of variants for a given position in a virus sample is close to equal, and the virus sequence cannot be identified unambiguously. Additionally, technical limitations may lead to sequencing uncertainties. As a result, some GenBank entries have ambiguous positions designated with IUPAC codes (Y for T/C, R for G/A, etc.). These data are not suitable for statistical phylogenetic analysis, and even a single ambiguous character in a large dataset can, under certain conditions, seriously disrupt the results by introducing a frameshift and compromising codon-position-sensitive substitution models. In most cases, such sequences can be omitted without compromising the study. For example, just 5% of GenBank *EV-A* sequences contain ambiguous characters. In other viruses, such as *FMDV*, 13.6% of sequences have ambiguous positions. If a single sequence has several ambiguous characters, this is likely the result of poor experimental data, and the sequence should be omitted. If single ambiguous characters are present in many sequences, or in particularly relevant sequences, they may be resolved to a consensus (the character found at this position in the most closely related sequences). This can be automated using the following algorithm (available at https://github.com/v-julia/resolve_ambiguous):

- Discard sequences with too many ambiguous characters, which may be a sign of poor sequence quality rather than natural heterogeneity (0.1%–0.2%, or one position in the full VP1, should have minimal effect on the analysis, see below);
- In the remaining sequences, identify a region (e.g., 100 nt) with an ambiguous character and blast it against the dataset;
- Identify the frequency of different nucleotides at this position in the most closely related sequences;
- Replace the ambiguous character with the most common nucleotide among the most closely related sequences.

Even a single artificial substitution can, in certain circumstances, affect the terminal branch rate upon a statistical phylogenetic analysis; therefore, if sequences with ambiguous characters are not particularly relevant for a study, it may be safer to omit them.

### 3.6. Effect of Sequencing Errors on Evolutionary Estimates 

Sequencing quality is not even across laboratories, and erroneous data could affect the outcome of phylogenetic analysis. In order to test the sensitivity of Bayesian phylogenetic methods to errors in virus sequences, we prepared simulated datasets that included sequences with artificially introduced substitutions. A dataset of all EV-A71 genotype B1 VP1 sequences (*n* = 56) was used. Five, 10, 15, or 20 random substitutions (simulated sequencing errors), or 0.56%, 1.1%, 1.7%, and 2.2% of sequence changes, respectively, were introduced into a single sequence (891 nt), and Bayesian phylogenetic analysis was performed. The analysis was done independently for a virus with a basal position on a tree (AB524092, a 1973 isolate from the Netherlands, hereafter termed “basal”) and an isolate from a dense superficial phylogenetic group with several closely related viruses (AF135866, a 1980 isolate from the USA, hereafter termed “treetop”).

**Figure 6:**
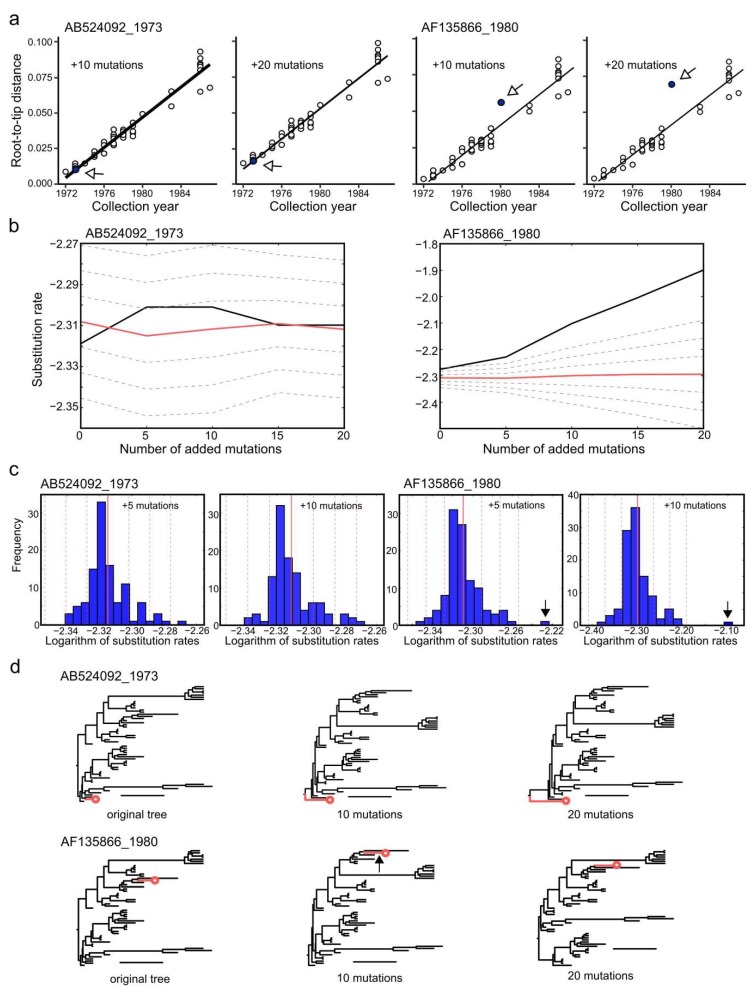
(**a**) The analysis of genotype B1 temporal structure using TempEst software after introducing 10 and 20 random mutations into sequences AB524092 and AF135866. Blue dots correspond to the isolates with the erroneous sequences. (**b**) The effect of adding random mutations on the substitution rates inferred in the Bayesian phylogenetic analysis for EV-A71 genotype B1 sequences AB524092 and AF135866. The mean rate in a tree is plotted with a bold red line; the terminal branch rate of the altered sequence is plotted with a black line. Dashed gray lines indicate mean ± 1SD, 2SD, and 3SD of the rates in the inferred trees. (**c**) The frequency distribution of decimal logarithms of substitution rates in genotype B1 trees that included sequences AB524092 and AF135866 after adding five random mutations. Solid and dashed red lines indicate mean rates and mean ± 1SD, 2SD, and 3SD, respectively. The arrow indicates the branch substitution rate for AF135866 after adding 5 and 10 random mutations. (**d**) The topology of maximum clade credibility (MCC) trees after adding random mutations to sequences AB524092 and AF135866. The number of added mutations is indicated below the tree. The branch leading to the sequence with simulated errors is colored in red and ends with a circle. White arrows show the positions of isolates with artificial mutations. The black arrows indicate the branch leading to AF135866 isolate, which had an aberrant rate on panel (**c**). Scale bars correspond to 5 years.

Analysis of the correlation of root-to-tip distances in phylogenetic tree and isolation dates is a classical approach to detecting abnormal evolutionary events. This analysis can demonstrate the temporal structure of a population, which has been recommended as a pre-requisite for statistical phylogenetic analysis [31]. Regression analysis with TempEst software could suggest erroneous sequences only in the case of the treetop sequence, and only 20 additional mutations resulted in an aberrant position of a point on the plot (Figure 6a). Bayesian phylogenetic analysis was then done for these simulated datasets with a single erroneous sequence. Tree branch rates were extracted from the resulting maximum clade credibility (MCC) trees using a Python script. As a relaxed lognormal molecular clock was used for phylogenetic inference, the rates had a lognormal distribution. To convert it into a normal distribution, which is more suitable for assessing extreme values, decimal logarithms of branch rates were used for further analysis. The effect of “sequencing errors” on the mean branch rate (red line), branch rate of the erroneous sequence (black line) and the standard deviation of branch rates across the tree (dashed lines) was plotted (Figure 6b). Sequencing errors had an almost negligible influence on the basal sequence, but a dramatic effect on both substitution rates across a tree and the individual rate of the treetop strain. Plotting the distribution of rates across the tree (Figure 6c) allowed for the clear identification of an erroneous treetop sequence with as few as five sequencing errors, or about 0.55%. 

The effect of sequencing errors on statistical phylogenetics may depend upon the position of a sequence and the presence of closely related sequences, among other factors and their combinations. At least in some cases, the rate distribution plot clearly indicated erroneous sequences, whereas regression analysis required arbitrary interpretation and was somewhat less sensitive (Figure 6a,c).

### 3.7. Effect of Annotation Errors on Evolutionary Estimates

**Figure 7:**
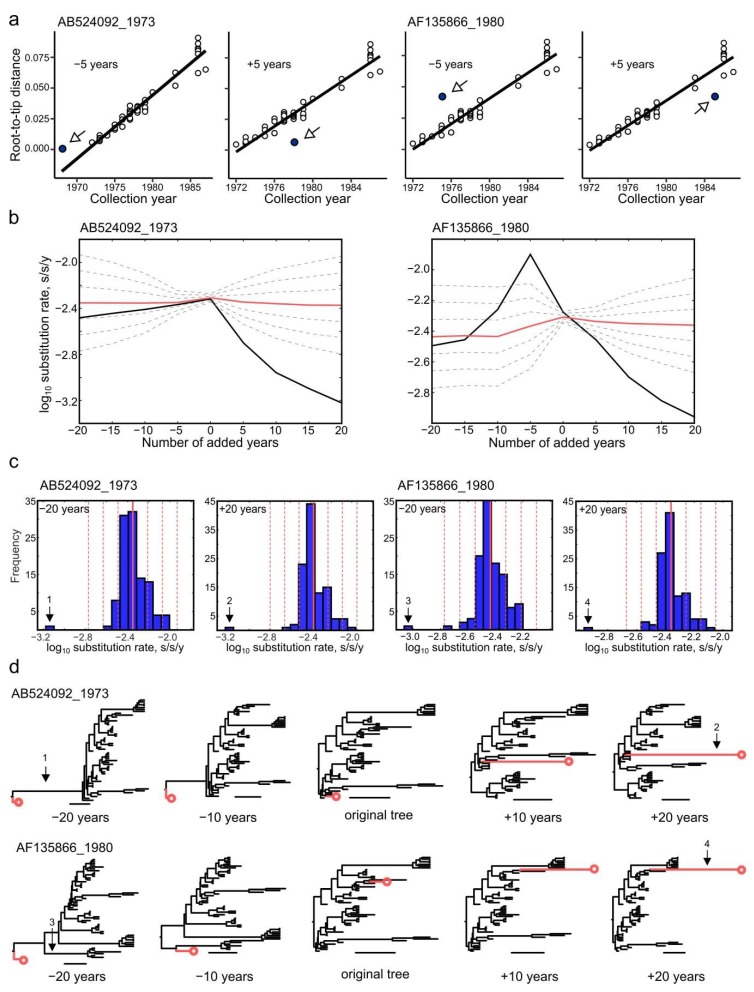
(**a**) Analysis of the EV-A71 genotype B1 temporal structure using TempEst software after adding and subtracting 5 years from the collection dates of either AB524092 or AF135866 isolates. Blue dots correspond to the isolates with an altered collection year. (**b**) The influence of changing the collection date of an isolate on the branch substitution rates inferred in Bayesian phylogenetic analysis for EV-A71 genotype B1 sequences AB524092 and AF135866. The mean branch rate is plotted with a bold red line; the terminal branch rate to the altered sequence is plotted with a black line. Dashed gray lines indicate mean ± 1SD, 2SD and 3SD of the branch rates in the inferred trees. (**c**) The frequency distribution of decimal logarithms of substitution rates in genotype B1 trees that included sequences AB524092 and AF135866 after adding or subtracting 20 years from their collection dates. Solid red and dashed grey lines indicate mean rates and mean ± 1SD, 2SD, and 3SD, respectively. (**d**) The topology of maximum clade credibility (MCC) trees (EV-A71, genotype B1) after adding and subtracting 10 and 20 years from the collection dates of isolates AB524092 and AF135866. The number of added years is indicated below the tree. The branch leading to the sequence with simulated errors is colored in red and ends with a circle. Scale bars correspond to 5 years. The numbered arrows indicate the branch substitution rates that exceed mean + 3SD and corresponding branches on the inferred MCC trees. White arrows show the position of isolates with altered collection year.

Bayesian phylogenetic methods rely on sequence isolation dates to infer evolution patterns over time. Simulated datasets with isolation dates of single EV-A71 genotype B1 sequences altered by 5, 10, or 20 years were created. As above, the analysis was done independently for a basal and a treetop sequence. An annotation error of 5 years from the actual date resulted in a notable deviation of the root-to-tip distance from the regression line for both basal and treetop sequences (Figure 7a, blue dots). As entries with a supposedly incorrect isolation date annotation are not uncommon in GenBank, correlation analysis and verification of the outlier sequences is strongly recommended prior to performing a statistical phylogenetic study. It is noteworthy that the correlation analysis can be much less sensitive on more heterogeneous datasets, such as the whole EV-A71 type dataset [55].

If sequences with incorrect isolation dates have not been excluded, they can significantly affect further analysis. Bayesian methods aim to infer the most likely phylogenetic history of the whole dataset under the chosen evolutionary model (substitution model, type of molecular clock, tree prior) and can smear the abnormalities caused by a single sequence over the whole system. Therefore, annotation errors of individual sequences may or may not affect the individual branch rates of the corresponding sequence (Figure 7b, black lines). However, even a minor tip date mistake of a single sequence has a dramatic effect on the standard deviations of branch rates across the whole tree (Figure 7b, dashed lines). For example, a modest error of 5 years increased the standard deviation of branch rates across a tree up to six times for a basal sequence and seven times for a treetop sequence. At the same time, a more significant annotation error (–20 years) did not cause a change in the erroneous branch rate, but rather increased the standard deviation of all branch rates across a tree by two times. Even if the individual branch rate estimates were not relevant for a given study, the increasing variance of parameters in a statistical model would lead to longer calculation times, which may be critical for large datasets. 

Even though the rate of the terminal branch of the erroneous sequence was not always affected, annotation errors resulted in the appearance of aberrant branches elsewhere in the tree (Figure 7c,d, indicated with arrows 1, 3). The effect of tip date errors on the overall tree topology can be gross, especially in the case of treetop sequences (Figure 7d). Therefore, careful selection of data, verification of sequences and isolation dates, and preliminary analysis of a dataset’s temporal structure can significantly improve the precision of statistical phylogenetic studies. 

## 4. Conclusions

Modern phylogenetic packages, such as BEAST, are relatively user-friendly and give simple access to advanced statistical methods. The selection of analysis parameters has been covered in detail in several studies [17,18,19,20]. Dataset preparation is usually given less attention. Many studies use arbitrarily selected sequences, fewer do systematic preparation, and just a few consider the artefacts that may be present in the GenBank data. Due to the nature of statistical phylogenetic systems, even minor errors in a small fraction of sequences may have a dramatic effect on the entire analysis. It is conceivable that, in an extreme case, a single erroneous sequence could render the analysis of several hundred sequences wrong, or even make it impossible. Unfortunately, there cannot be fixed rules for dataset preparation. Regression analysis of isolation time and root-to-tip distance allows for the identification of date annotation errors and is necessary in the case of enteroviruses and other picornaviruses. The distribution of branch rates (or their logarithms) of the MCC tree should be close to normal for most picornaviruses, due to their biological properties, and is a good way to detect unnatural events, such as annotation errors or the release of an archive virus [55]. The criteria for the exclusion of sequences remain arbitrary and should consider many factors, especially because certain natural events, such as rapid re-adaptation of viruses to a new system [56] or long-term persistence [57], can result in aberrant rates in a virus lineage. Therefore, dataset preparation is a crucial step for picornavirus phylogenetic studies, and probably requires even more attention than the optimization of the statistical analysis parameters. 

## Figures and Tables

**Table 1:** Substitution rates in picornaviruses.

Virus	Rate, ×10^−3^ s/s/y	Reference
*Hepatitis A virus*	1.0	[3]
	1.21–2.0	[4]
	0.6	[5]
*Duck Hepatitis A Virus*	0.6–1.9	[6]
*FMDV*	serotype O–6.0 serotype A–11.9 serotype Asia-1–3.1	[7]
	serotype Asia1–5.9	[8]
	serotype SAT1–3.00 serotype SAT2–4.0	[9]
*Parechovirus*	2.8	[10]
Non-polio enteroviruses	6.0–11.0	[11]
	3.40–11.9	[12]
Enterovirus A71	3.6–5.3	[13]
	4.2–4.6	[14]
Poliovirus	10.0	[15]
*Teschovirus A*	1.62	[12]
	2.46	[16]
*Cardiovirus A*	1.61	[12]
